# Impact of Heat and Cold on Total and Cause-Specific Mortality in Vadu HDSS—A Rural Setting in Western India

**DOI:** 10.3390/ijerph121214980

**Published:** 2015-12-02

**Authors:** Vijendra Ingole, Joacim Rocklöv, Sanjay Juvekar, Barbara Schumann

**Affiliations:** 1 Vadu Rural Health Program, KEM Hospital Research Centre, Pune 411011, India; sanjay.juvekar@gmail.com; 2 Epidemiology and Global Health, Department of Public Health and Clinical Medicine, Umeå University, Umeå 901 87, Sweden; joacim.rocklov@umu.se (J.R.); barbara.schumann@umu.se (B.S.); 3 INDEPTH Network, Accra KD 213, Ghana; 4 Centre for Demographic and Ageing Research, Umeå University, Umeå 901 87, Sweden

**Keywords:** heat, cold, temperature, mortality, cause-specific mortality, India

## Abstract

Many diseases are affected by changes in weather. There have been limited studies, however, which have examined the relationship between heat and cold and cause-specific mortality in low and middle-income countries. In this study, we aimed to estimate the effects of heat and cold days on total and cause-specific mortality in the Vadu Health and Demographic Surveillance System (HDSS) area in western India. We used a quasi-Poisson regression model allowing for over-dispersion to examine the association of total and cause-specific mortality with extreme high (98th percentile, >39 °C) and low temperature (2nd percentile, <25 °C) over the period January 2003 to December 2012. Delays of 0 and 0–4 days were considered and relative risks (RR) with 95% confidence intervals (CI) were calculated. Heat was significantly associated with daily deaths by non-infectious diseases (RR = 1.57; CI: 1.18–2.10). There was an increase in the risk of total mortality in the age group 12–59 years on lag 0 day (RR = 1.43; CI: 1.02–1.99). A high increase in total mortality was observed among men at lag 0 day (RR = 1.38; CI: 1.05–1.83). We did not find any short-term association between total and cause-specific mortality and cold days. Deaths from neither infectious nor external causes were associated with heat or cold. Our results showed a strong and rather immediate relationship between high temperatures and non-infectious disease mortality in a rural population located in western India, during 2003–2012. This study may be used to develop targeted interventions such as Heat Early Warning Systems in the area to reduce mortality from extreme temperatures.

## 1. Introduction

Global climate change is likely to increase the frequency and intensity of heat waves [1]. Humans are exposed to climate change through changing weather patterns (temperature, precipitation, and more frequent extreme events). Heat related mortality and morbidity can occur through direct and indirect pathways, e.g., through heat exhaustion or heat stroke, renal insufficiency, acute cerebrovascular disease and exacerbations of pulmonary disease [2,3,4]. According to the International Disaster Database (EM-DAT), in India, 10,389 deaths were caused by extreme high and low temperature between January 1990 and March 2014 [5]. Evidence shows that heat waves are associated with high and excess mortality especially in rural populations, among the elderly and outdoor workers in India and South Asia [6,7]. In a previous study, we have shown that mortality is affected by both increased and decreased temperatures and rainfall in the age groups of children (0–5 years) and adults aged 20–59 years within a shorter lag period up to two weeks [8]. Researchers reported that in Bangladesh impacts of high and low temperature were larger on the infant and elderly population (aged 60 years and older) [9]. There have been very few studies so far investigating cause-specific mortality in association with heat events in low and middle-income countries [3,9,10,11]. In the present study, we aimed to investigate heat and cold effects on total mortality, infectious disease mortality, non-infectious diseases mortality and external causes of death between 2003 and 2012 using data of the Vadu Health and Demographic Surveillance System (HDSS) in western India.

## 2. Methods

### 2.1. Study Area and HDSS Setting

**Figure 1:**
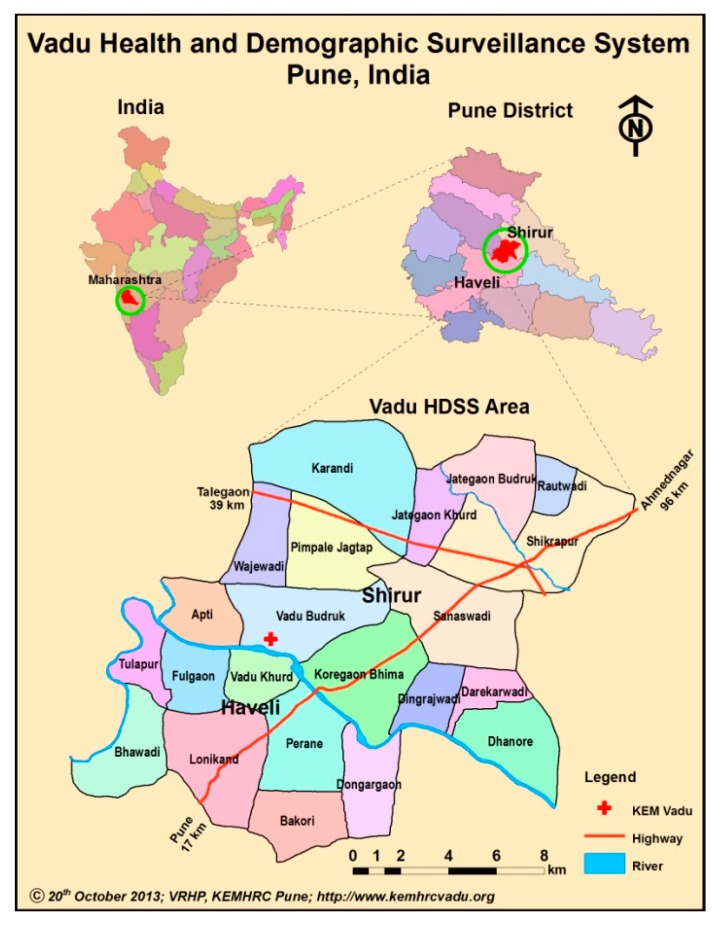
Map of Vadu HDSS area (22 villages of Pune district) located in Western India.

Vadu HDSS is a member center of the International Network for the Demographic Evaluation of Populations and Their Health (INDEPTH) in low and middle-income countries. The INDEPTH Network is a global alliance including 52 HDSS field sites in 45 research centers in Asia, Africa and Oceania [12]. Vadu HDSS covers 22 villages from two administrative blocks in the rural Pune district of Maharashtra state, in western India (Figure 1). The total population was 131,545 (Vadu HDSS Database, August 2012). The geographical extent is 18°30ʹ to 18°47ʹ N Latitude and 73°58ʹ to 74°12ʹ E Longitude, covering approximately 232 km^2^.

### 2.2. Mortality and Verbal Autopsy Data

The study samples were obtained from Vadu HDSS for the period of January 2003 to December 2012. In Vadu HDSS, Field Research Assistants (FRAs) visit each household and record demographic events (births, deaths, in-migration, out-migration, pregnancies) twice a year. All deaths within the study area are recorded and subjected to verbal autopsy (VA). The verbal autopsy is an instrument for identifying the cause of death on the basis of structured interviews with relatives of the deceased after the death has occurred. Information obtained in these interviews is used to determine the likely cause of death [13,14]. Trained field research assistants administered the VA tool to all deaths occurring in the Vadu HDSS area. These data were collected within four weeks after the death occurred.

In this analysis, we have used data of 2302 deaths for which verbal autopsy was performed (population aged 12 years or older). For each deceased, age, gender, cause of death, and date of death were obtained. International classification of Diseases (ICD)-10 codes were assigned by a physician and were grouped into four major classes of diseases: infectious diseases (ICD A00-B99, J00-J99, L00-L08, M00-M03), non-infectious diseases (ICD C00-D48, E00-E90, F00-F99, G00-G99, H00-H95, I00-I99, K00-K93, N00-N99), external causes of death (ICD S00-T98, V01-Y98), and unspecified causes of death (ICD R00-R99; not considered in the analysis).

### 2.3. Weather Data

Daily maximum and minimum temperature data at the Pune Airport station (15 km southwest of the study area) were acquired from the Indian Meteorological Department (IMD) for the study period of January 2003 to December 2012. We used daily maximum temperature due to the fact that this variable had the least number of missing observations and errors.

### 2.4. Definition of Heat Days and Cold Days

This study adopted a percentile approach to identify hot days and cold days [15,16,17,18]. There is no standard definition of heat and cold with regard to health impacts [19]. A definition of heat wave and cold wave always considers the combination of intensity and duration of a high temperature period [20,21,22]. In the present study, heat days were defined as days with maximum temperatures above the 98th percentile (>39 °C), and cold days as days with maximum temperatures below the 2nd percentile (<25 °C).

### 2.5. Statistical Analysis

We used a quasi-Poisson regression model to examine the association of heat and cold days with daily counts of total and cause-specific deaths, respectively. First, we estimated the relationship between heat and cold days with mortality in lag 0 (same day) and lag 0–4 days (any day of heat/cold during the last five days). Models were adjusted for day of week; natural cubic splines for time with equally arranged knots were used to control for secular trends and for other time-varying confounding factors. Five degrees of freedom per year for ten years were used in the model. The mathematical expression of the model is given as follows:

Log(Y)=β0+β1Χi+ns(time,5df/year)+other covariates

where *Y* is occurrence of deaths, β0 is the intercept, β1 regression coefficient for the indicator variable Χi marking a heat or cold day or cumulative days of heat wave or cold wave (lag 0–4 days) and ns describes patterns over time and is a natural cubic spline function with 5 degrees of freedom per year of study, and other covariates include weekdays. Relative risks (RR) with 95% confidence intervals (CI) were computed.

Additionally, we assessed in sensitivity analyses the relationship between heat and cold days and daily deaths using a logistic regression model to check the robustness of the quasi-Poisson model given the large number of days with zero deaths (Table A1). This analysis was done using the statistical software R-version 3.1.0.3.

## 3. Results

Table 1 presents the distribution of daily maximum temperature; Table 2 shows descriptive summary statistics of daily total and cause-specific deaths during the study period January 2003 to December 2012. The largest disease group was non-communicable diseases, mainly consisting of acute myocardial infarction, stroke, actual renal failure, asthma, and chronic ischaemic heart disease. Deaths by infectious diseases include gastroenteritis, amoebiasis and sepsis as the most frequent causes of death. Unspecified injuries, intentional self-harm and accidents were the main external causes of deaths.

**Table 1:** Daily Maximum Temperature (°C), 2003–2012.

Minimum	Maximum	Mean	Median	2nd Percentile	5th Percentile	95th Percentile	98th Percentile
21.1	42.4	32.0	31.2	25.4	26.6	38.8	39.9

**Table 2:** Causes of death at Vadu HDSS, 2003–2012.

Causes of Death	N	%
Non-infectious diseases	1175	51.04
Infectious diseases	296	12.86
External causes	309	13.42
Unspecified causes	522	22.68
Total deaths	2303	100

**Figure 2:**
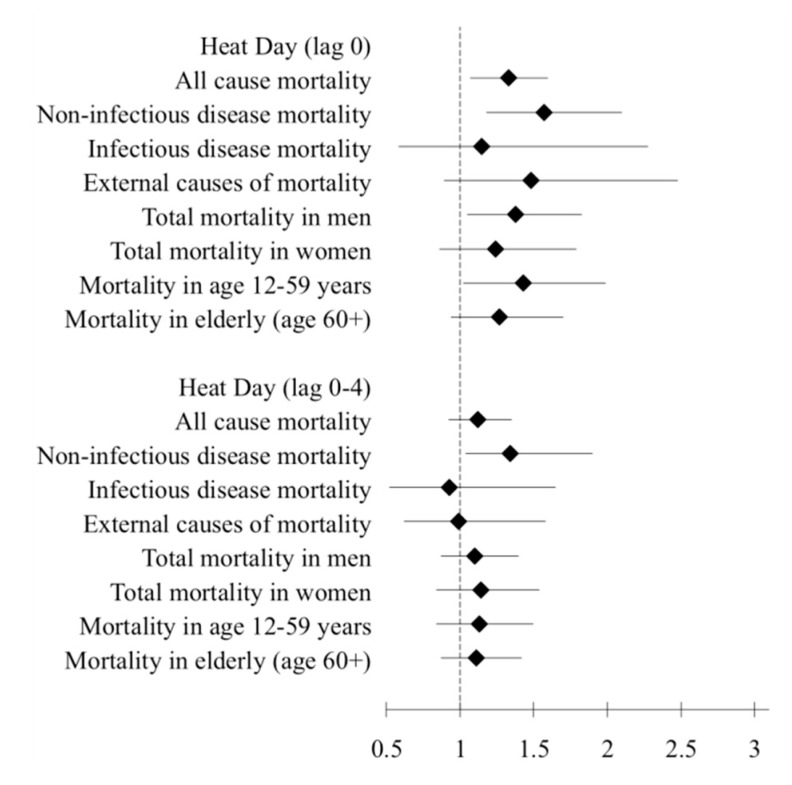
Association of heat (98th percentile, >39 °C) with total, cause-specific, age and sex-specific mortality in Vadu HDSS 2003–2012.

**Figure 3:**
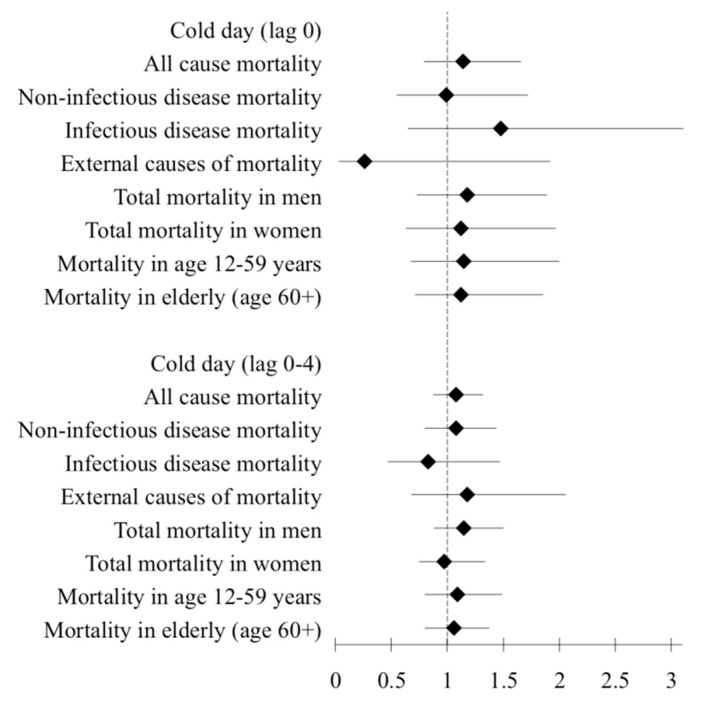
Association of cold (2nd percentile, <25 °C) with total, cause-specific, age and sex-specific mortality in Vadu HDSS 2003–2012.

There were in total 144 heat days and 44 cold days over the 10 years period. Relative risks for total and cause specific mortality with 95% confidence intervals are presented in Figure 2 and Figure 3 (supplementary Table S2).

In lag 0, we observed that heat days (98th percentile, >39 °C) were associated with an increased total mortality (RR = 1.33; CI: 1.07–1.60), *i.e.*, an increase by 33%. There was a statistically significant association between heat and mortality from non-infectious diseases on the same day (RR = 1.57; CI: 1.18–2.10) as illustrated in Figure 2. Also in longer lags (0–4 days), we found that non-infectious disease mortality was associated with heat days (RR = 1.34; CI: 1.04–1.90). Infectious disease mortality and external causes of death were not associated with heat days. The relative risk of total mortality in men on heat days (lag 0) was 1.38 (CI: 1.05–1.83); however, no statistically significant effect was found in women. Mortality in age group 12–59 years showed RR=1.43 (CI: 1.02–1.99) on the same day (lag 0) of heat. We did not find any significant association between total or cause-specific mortality and cold days (2nd percentile, <25 °C) over lag 0 and 0–4 days (Figure 3).

In sensitivity analyses using a logistic regression model, we found similar results as with the quasi-Poisson model. However, effects were marginally higher than those of the quasi-Poisson regression model for heat days. For heat we found increases of 70% and 44% in non-infectious disease mortality for lag 0 and lag 0–4 days respectively. Logistic regression models too, retrieved non-significant results for the impact of cold days for lag 0, lag 0–4 and lag 0–14 days (Table A1).

## 4. Discussion

To the best of our knowledge, this is the first study in a rural population of India to assess the relationship between extreme high and low temperature and total mortality and cause-specific mortality using verbal autopsy data.

We found that total mortality and deaths by non-infectious diseases are associated with heat days. There was less evidence for an effect of heat days on total mortality among women and elderly in this population. Relative risks among men and age group 12–59 years were higher than in women and elderly, and statistically significant. We did not find any association of cold days and mortality by cause of death, sex and age group. Firstly, we observed a strong significant immediate impact of high temperature (57% increase) on deaths by non-infectious diseases. In fact, heat effects on non-communicable disease mortality in our population were larger on the same day than in the lag period 0–4 days. There is evidence that not only heat strokes, but also cardiac disease, and renal impairment are associated with heat [23]. The population in Vadu HDSS was highly affected by non-infectious disease, such as cardiovascular diseases (e.g., cardiac arrest, myocardial infarction), respiratory diseases (specifically asthma) and kidney disease (acute renal failure).

Secondly, total mortality was 33% higher during heat events. The present findings seem to be consistent with other research, which found a strong relationship between high daily temperature and all cause-mortality in South East Asia. For India, immediate and lagged effects of high temperature on mortality were reported [7,8].

Thirdly, our results show that heat was associated with a 43% increase of deaths in the age group 12–59 years. Previous research suggested that heat effects were mainly found in elderly and women [24]. Fourthly, we observed a contrasting heat mortality relationship between men and women, with men aged 12–59 years of age experiencing higher relative risk. This may be explained by the fact that in our population, more men are working in agriculture and manufacturing industry. Research in developed countries showing that work in agriculture and construction increase the risk of heat-associated mortality [25,26]. From an international perspective, the comparatively large relative risks observed during heat events suggest the rural population may be particularly susceptible to heat. It may also reflect either a lack of awareness or limits to adaption to heat exposure in the labor active population, may be through economic incentives, causing abnormal metabolic heat production followed by disease and death.

We did not find any impact of heat on mortality by infectious diseases and external causes. This result is not consistent with studies from developed countries, which have demonstrated that mortality by respiratory diseases and external causes was strongly associated with hot weather [27]. However, the non-significant results on external causes of deaths may be related to limited statistical power in this study.

Prior research suggests that high and low temperatures have different lag effects. Generally, effects of cold have longer delays than of hot days [28,29]. However, we assessed in sensitivity analyses lags up to 14 days and found non-significant results (supplementary material Table A1 and Table A2), thus, there was no impact of cold on mortality in our study population. A reason for this may be the threshold for cold, defined as temperatures below 25 °C (2nd percentile), which is relatively high compared to studies from temperate countries. 

In the future, the Vadu population may become more vulnerable to heat effects due to economic development or unplanned rapid growth. A growing population can place a burden on sanitation, air pollution and overcrowding, which may increase future human vulnerability to climate change [30,31]. Different measures including health advice and weather based warning systems can be applied to prevent heat-related deaths. Furthermore, population adaptive capacity needs to be improved in the Vadu HDSS area to cope with extreme weather effects. However, there is no clear evidence about the most effective measures when targeting vulnerable groups [3]. There is a great need for adaptation planning in rural areas of developing countries, which have been largely overlooked to date.

A key policy priority of local governments should therefore be the development of appropriate response plans to cope with the increasing threat from heat waves [32]. Moreover, public awareness about the effect of heat on health is also critical to saving lives. Awareness messages must be simple and easy to understand by the local population. These messages may include recommendations to check weather forecasts, avoid physical activity and work in the hottest period of the day and target particularly vulnerable people in the community [7]. Effective measures for prevention policies require understanding of particular work related, demographic, social, and ecological determinants and sensitivities of the population [7]. More research is needed to improve understanding of the modulating factors such as housing quality, technology, local topography, urban design, and behavioral factors [30].

Therefore, future studies should focus on understanding causal pathways between weather extremes, particularly heat waves, and health outcomes by assessing socio-demographic parameters.

### Strengths and Limitations of the Study

Our study has some strengths and limitations that should be noted. One of the strengths of this study is the verbal autopsy data (high precision), commonly not available in low and middle income countries. In this analysis, we have considered only those deaths for which verbal autopsy has been done, including only those aged 12 years and older. Results might be, different for younger children. In many studies, humidity, apparent temperature and air pollution are used as confounding factors, but we did not have this data for our study area [15,33]. However, adjustment for such confounding factors should be carefully motivated and rather an exception than a rule as has been illustrated using directed acyclic graphs to illustrate the causal processes included in the linkage between the exposures [34]. We also observed a large number of days with zero deaths, which limits statistical power. Another limitation is that the location of the weather station was at some distance from the actual study area, which might be a reason for underestimation of true associations.

## 5. Conclusions

In this investigation, we assessed the relationship between heat and cold days and total and cause-specific mortality in a rural part of India. The study concludes that there is an immediate association of high temperature and non-infectious disease mortality. One of our study findings suggests that men of working age were more vulnerable to heat, which suggests to focus on specific groups e.g., agricultural and industrial worker who are at higher risks. Therefore, further studies should look into socio-economic and occupational groups in the population for targeting interventions. This information can be used to develop targeted interventions such as a heat early warning system in the study area and health interventions to prevent heat-related deaths in the near future.

## Appendix

**Table A1:** Association of heat and cold with mortality stratified by cause, age, and sex Vadu HDSS, 2003–2012 (odds ratios with 95% confidence intervals).

**Heat Days**	**Lag 0**		**Lag 0–4 Days**	
	**OR**	**95% CI**	**OR**	**95% CI**
All cause mortality	1.40	0.95–2.06	1.17	0.87–1.59
Non-infectious disease mortality	**1.70**	**1.13–2.54**	**1.44**	**1.03–2.00**
Infectious disease mortality	1.21	0.55–2.42	0.96	0.51–1.73
External causes of death	1.75	0.96–3.06	1.10	0.65–1.84
All cause mortality in men	1.10	0.72–1.65	1.02	0.73–1.42
All cause mortality in women	1.12	0.70–1.77	1.12	0.77–1.61
All cause mortality in age group 12–59 years	1.42	0.92–2.17	1.19	0.84–1.70
All cause mortality in age group 60+ years	1.34	0.88–2.01	1.34	0.88–2.01
**Cold Days**	**Lag 0**		**Lag 0–4 days**	
	**OR**	**95% CI**	**OR**	**95% CI**
All cause mortality	1.71	0.89–3.32	1.26	0.88–1.81
Non-infectious disease mortality	0.87	0.39–1.78	1.11	0.75–1.64
Infectious disease mortality	1.72	0.62–4.05	0.89	0.46–1.60
External causes of death	0.26	0.03–1.95	1.21	0.66–2.22
All cause mortality in men	1.48	0.75–2.82	1.24	0.85–1.79
All cause mortality in women	0.88	0.38–1.84	0.97	0.63–1.46
All cause mortality in age group 12–59 years	1.37	0.69–2.72	1.28	0.87–1.88
All cause mortality in age group 60+ years	1.35	0.67–2.62	1.04	0.70–1.52
**Cold Days**	**Lag 0–14**			
	**OR**		**95% CI**	
All cause mortality	1.07		0.82–1.39	
Non-infectious disease mortality	1.08		0.81–1.44	
Infectious disease mortality	0.92		0.59–1.44	
External causes of death	0.73		0.44–1.18	
All cause mortality in men	0.99		0.75–1.31	
All cause mortality in women	1.08		0.80–1.46	
All cause mortality in age group 12–59 years	1.13		0.84–1.52	
Total mortality in age group 60+ years	1.04		0.78–1.37	

Logistic regression model (OR, odds ratios; CI, confidence interval; bold numbers indicate statistical significance (*p* < 5%).

**Table A2:** Association of heat and cold with mortality stratified by cause, age, and sex Vadu HDSS, 2003–2012 (relative risks with 95% confidence intervals).

**Heat Days**	**Lag 0**		**Lag 0–4 Days**	
	**RR**	**95% CI**	**RR**	**95% CI**
All cause mortality	**1.33**	**1.07–1.60**	1.12	0.92–1.35
Non-infectious disease mortality	**1.57**	**1.18–2.10**	**1.34**	**1.04–1.90**
Infectious disease mortality	1.15	0.58–2.28	0.93	0.52–1.65
External causes of mortality	1.48	0.89–2.48	0.99	0.62–1.58
All cause mortality in men	**1.38**	**1.05–1.83**	1.10	0.87–1.40
All cause mortality in women	1.24	0.86–1.79	1.14	0.84–1.54
All cause mortality in age group 12–59 years	**1.43**	**1.02–1.99**	1.13	0.84–1.50
All cause mortality in age group 60+ years	1.27	0.94–1.70	1.11	0.87–1.42
**Cold Days**	**Lag 0**		**Lag 0–4 days**	
	**RR**	**95% CI**	**RR**	**95% CI**
All cause mortality	1.14	0.79–1.66	1.08	0.87–1.32
Non-infectious disease mortality	0.99	0.55–1.72	1.08	0.80–1.44
Infectious disease mortality	1.48	0.65–3.38	0.83	0.47–1.47
External causes of mortality	0.26	0.03–1.92	1.18	0.68–2.06
All cause mortality in men	1.18	0.73–1.89	1.15	0.88–1.50
All cause mortality in women	1.12	0.63–1.97	0.97	0.75–1.34
All cause mortality in age group 12–59 years	1.15	0.67–2.00	1.09	0.80–1.49
All cause mortality in age group 60+ years	1.12	0.71–1.86	1.06	0.80–1.38
**Cold Days**	**Lag 0–14**			
	**RR**		**95% CI**	
All cause mortality	1.01		0.86–1.18	
Non-infectious disease mortality	1.08		0.87–1.33	
Infectious disease mortality	0.88		0.59–1.33	
External causes of death	0.72		0.46–1.14	
All cause mortality in men	0.98		0.80–1.21	
All cause mortality in women	1.03		0.82–1.30	
All cause mortality in age group 12–59 years	1.03		0.82–1.29	
All cause mortality in age group 60+ years	0.99		0.81–1.21	

Poisson regression model (RR, relative risk; CI, confidence interval; bold numbers indicate statistical significance (*p* < 5%).
